# A methodological approach to identify the most reliable human milk collection method for compositional analysis: a systematic review protocol

**DOI:** 10.1186/s13643-018-0788-4

**Published:** 2018-08-16

**Authors:** Gabriela E. Leghi, Philippa F. Middleton, Beverly S. Muhlhausler

**Affiliations:** 1 Food and Nutrition Research Group, Department of Food and Wine Sciences, School of Agriculture, Food and Wine, Waite Campus, The University of Adelaide, Urrbrae, South Australia 5064 Australia; 2 Healthy Mothers, Babies and Children Theme, South Australian Health and Medical Research Institute (SAHMRI), Adelaide, 5001 Australia

**Keywords:** Systematic review, Human milk composition, Breast milk collection, Infant health

## Abstract

**Background:**

Breast milk composition has been reported to vary significantly between individual women and between different populations. However, the composition is also known to vary within the same woman between different days, within the same day, and even across the same feed. Therefore, it is unclear to what extent variations in composition are due to variations in sampling methodology between studies. The purpose of this systematic review is to compare the results obtained for breast milk macronutrient composition between studies utilizing different sampling methodologies and to use this as a basis to determine the most robust and consistent sampling approach as an alternative to full expression (gold standard).

**Methods:**

The EMBASE, MEDLINE/PubMed, Cochrane Library, Scopus, Web of Science, and ProQuest Dissertations and Theses Global databases will be searched for relevant articles. Observational studies, including cross-sectional, comparative cohort, and longitudinal cohort studies which involve lactating women who are breastfeeding (exclusively or not) or expressing (manually or using a breast pump) at any lactation stage will be included. This review will compare different methods of breast milk collection used in research studies which report macronutrient levels (protein, fat, lactose). Two review authors will independently screen titles and abstracts of studies identified by the literature search to determine articles for the full text screening. Quality assessment of included articles will be conducted independently by two review authors using the Newcastle-Ottawa scale.

**Discussion:**

It is important to identify the most reliable and practical method of human milk collection which best represents the average composition of the milk that is being consumed by the infant. This systematic review will be critical for ensuring that we determine a robust and consistent sampling approach to use in future studies of evaluating breast milk composition in a larger population. Identifying a recommended standard collection protocol will also provide more opportunities for sharing and combining data from different research groups, thus enhancing replicability and knowledge in the field.

**Systematic review registration:**

PROSPERO CRD42017072563

**Electronic supplementary material:**

The online version of this article (10.1186/s13643-018-0788-4) contains supplementary material, which is available to authorized users.

## Background

Breast milk is uniquely designed for the human infant; it is thought to contain adequate nutrients and bioactive components and is known for its important role in infant survival and health [1]. The World Health Organization (WHO) recommends that infants should be exclusively breastfed until around 6 months’ postpartum, with continued breastfeeding to 12 months of age and beyond [2]. It is critical to understand contemporary breast milk composition given the established importance of nutritional exposures in early infancy for an individual’s life-long health outcomes; a concept that has been reinforced by reports of significant relationships between the concentration of specific components in breast milk and infant growth/body composition and future risk of obesity [3].

Despite recognition of the importance of breastfeeding and human milk, however, research in this area is complicated by the fact that human milk composition is highly variable and can change according to time since last feed, period of the day, and stage of lactation, as well as between women and populations [4]. The method and time of sample collection has been shown to influence breast milk composition, including the levels of macronutrients (fat, protein, and lactose), micronutrients, as well as levels of other bioactive factors in breast milk [4]. For example, the milk fat content is lower at the beginning of the feed compared with the end of the feed and can increase up to three times from its initial concentration across a single feeding period [5].

Several studies have suggested that the “gold standard” method of sampling human milk for compositional analysis is to collect small samples from multiple complete breast expressions breastfeed across a 24-h period [4, 6]. However, this approach is impractical for many women, particularly those who have a limited milk supply and/or whose infants refuse to consume expressed breast milk from a bottle, and is therefore less than ideal for studies in which the aim is to assess breast milk composition in a large population.

There are very few studies which have systematically evaluated the full range of breast milk components collected at different times during a feed and across a 24-h period and compared these to those obtained using this “gold standard” methodology.

### Aim

The purpose of this systematic review is to compare the results obtained for breast milk macronutrient composition between studies utilizing different sampling methodologies and to use this as a basis to determine the most robust and consistent sampling approach as an alternative to full expression (gold standard).

## Methods

### Protocol

The present systematic review protocol has been developed based on Preferred Reporting Items for Systematic Reviews and Meta-Analysis Protocols (PRISMA-P) guidelines [7, 8] and Cochrane Handbook for Systematic Reviews of Interventions [9]. A PRISMA-P checklist file is attached (Additional file 1).

### Eligibility criteria

Studies will be selected according to the criteria outlined below. Studies only reported as abstracts will not be included.

#### Study design

All observational studies, including cross-sectional, comparative cohort, and longitudinal cohort studies will be included.

#### Participants

Included studies will involve women who are breastfeeding (exclusively or partially) or expressing (manually or using a breast pump) at any lactation stage.

#### Method of collection

To be eligible for inclusion, studies must report the time of day of collection, state the method of collection, and whether collection was undertaken by manual expression, a breast milk pump (or either), or any other method.

#### Comparators

Comparison of average macronutrient composition obtained in studies using the gold standard collection methods (full expression of a breast or both breasts, or subsamples from all full expressions, across a 24-h period) vs other breast milk collection methods

#### Outcomes

Breast milk macronutrient composition (total protein, total fat, and lactose)

#### Timing

Any time during lactation

#### Setting

There will be no restrictions on the type of setting.

#### Language

Only articles reported in the English language will be included due to lack of resources for translation.

### Information sources

Literature searches will be undertaken using the following electronic bibliographic databases: EMBASE, MEDLINE/PubMed, Cochrane Library, Scopus, Web of Science, and ProQuest Dissertations and Theses Global. The literature search will be limited to studies in humans, but no date range restrictions will be applied. For each selected article, abstracts and full articles will be obtained. In order to increase literature coverage, we will also scan the reference lists of included studies and systematic reviews identified during the screening process.

### Search strategy

The specific search strategies will be developed by the research team with the input from an experienced research librarian. The literature search will be updated towards the end of the review, just prior to final analysis, to ensure maximum coverage of eligible studies. A draft search strategy for MEDLINE, including search terms, is included in Additional file 2. After the MEDLINE search is complete, we will adapt the search strategy to the subject headings and syntax of the other electronic databases.

### Study records

#### Data management

The literature search will be initially performed using the selected databases and then uploaded into EndNote software [10], a reference management software that allows organization of references identified from different electronic databases. All search results will be entered into a single EndNote library, and duplicate studies will be identified and removed. After removal of duplicates, the search results will then be uploaded to Covidence [11], an Internet-based software program that facilitates article screening, data extraction, and collaboration among multiple reviewers. The team will develop and test screening questions and forms based on the eligibility criteria. Citation titles and abstracts will be uploaded to the software.

#### Selection process

The selection of articles for inclusion in the review will be undertaken in two stages. The first stage will involve screening the title and abstracts of the search results against the eligibility criteria. In the second stage, the full articles of these articles will be screened to confirm that they meet the eligibility criteria. At both stages, each article will be screened independently by two authors. Any articles where there is disagreement in eligibility status between the first two authors will be reviewed by the third author and any disagreements resolved by mutual discussion. The reviewers will not be blinded to journal of publication or authors and institutions at any stage of the screening process. Reasons for excluding studies will be documented and reported, and the number of articles and reasons for exclusion at each stage of screening will be presented in a Preferred Reporting Items for Systematic Reviews and Meta-Analysis (PRISMA) flow diagram.

#### Data collection process

Two authors will independently extract data from each included study (which may have one or more publications) based on a standardized extraction form from the Cochrane Pregnancy and Childbirth group [12]. All authors will review and discuss the data extraction form prior to commencing data extraction, and all authors will complete and compare data extraction from a single selected study to ensure that there is consistent interpretation of the questions. To minimize inconsistency between reviewers, we will conduct training and calibration exercises using the data extraction form prior to the commencement of the systematic review.

Data extracted will include study design, sample size, number of milk samples analyzed, and methodological details about breast milk collection including:

(i) Whether or not the women were exclusively breastfeeding
(ii) Lactation day/stage when breast milk was collected (time postpartum)
(iii) Time of day milk was expressed/collected
(iv) Time since last feed/expression that milk samples were collected
(v) Whether separate pre- or post-feed/expression samples were collected or milk samples were mixed
(vi) Any details provided as to which breast was used for collection (e.g., some authors discuss the letdown response in the absence of suckling, so they ask women to breastfeed baby at one breast while collecting milk from the other)
(vii) Whether milk was expressed manually or using a breast pump—duration of pumping and milk volume expressed will also be reported
(viii) If a pump was used, then the type of pump
(ix) Volume of milk consumed by infant (if samples collected at time of a feed)
(x) Gestational age of infant at birth (e.g., preterm or term)

The data extraction form will be completed by at least two authors for each study. One author will be responsible for cross-checking each data extraction form for completeness. Any differences in the data extracted from a particular study will be resolved by discussion between the two reviewing authors, and in the case of a disagreement between these authors, reviewed by a third author. If missing data are identified, then reasonable efforts will be made to contact the corresponding authors of the relevant study by email (maximum three attempts) to obtain this missing information.

### Risk of bias in individual studies

Quality assessment of each included articles will be conducted independently by two authors based on the Newcastle-Ottawa scale (NOS) which was designed specifically for assessment of non-randomized studies, including cohort studies [13], and adapted for cross-sectional studies. The information captured in the NOS will be used to guide the allocation of studies as being at either low or high risk of bias (or unclear), based on criteria such as attrition rate, sample size, and risk of confounding. In addition to the overall analysis of findings from all selected studies, we will also consider the findings from studies with low and high/unclear risk of bias separately to determine the potential influence of study quality on the overall conclusions.

### Data synthesis

The extracted data will be presented as structured summary tables which include a description of study characteristics, including number of participants, timing (stage of lactation), type of breast milk collection, and results of macronutrient analyses (total protein, total fat, and/or lactose). Risk of bias for each study will also be included. The findings from different studies in relation to macronutrient concentrations (total fat, total protein, and/or lactose) will be summarized and compared between studies utilizing different methods of breast milk collection.

## Discussion

### What is the issue?

There is currently a lack of consensus on the most reliable alternative method of human milk collection to the subsampling and pooling of milk samples from a full breast expression at each feed across a 24-h period. While this method is considered the “gold standard,” it is invasive and impractical for population studies in which the goal is to obtain compositional information on a representative sample of women. Currently, an array of alternative collection methods is utilized by different research groups, and there is no universal/standardized sampling approach.

### Why is this important?

It is important to identify the most reliable and practical method of human milk collection which will best represent the average composition of the milk that is being consumed by the infant. Breast milk is known to vary in composition across the day and during an individual feed, but to date, it is unclear whether one particular collection method is more closely aligned to the “gold standard” approach of sampling from all full breast milk breastfeeds/expressions collected across a 24-h period. This systematic review will also compare the results obtained for macronutrient composition using different collection methods. Ultimately, the outcomes of this systematic review will identify a recommended standard collection protocol for use by all researchers working in human milk research, with the potential to significantly expand the opportunities for sharing and combining data from different research groups. This will enhance replicability and knowledge in the field.

## Additional files

Additional file 1: PRISMA-P checklist. (DOCX 31 kb)

Additional file 2: Example Search Strategy - MEDLINE/PubMed. (DOCX 19 kb)

## Abbreviations

NOS: Newcastle-Ottawa scale
PRISMA: Preferred Reporting Items for Systematic Reviews and Meta-Analysis
PRISMA-P: Preferred Reporting Items for Systematic Reviews and Meta-Analysis Protocols
WHO: World Health Organization
