# Supplementary material for: A methodological approach to identify the most reliable human milk collection method for compositional analysis: a systematic review protocol
Source: Syst Rev. 2018 Aug 16;7:122. doi: 10.1186/s13643-018-0788-4 (PMC6097334; doi:10.1186/s13643-018-0788-4)
Supplement: Supplementary file 2 — Example Search Strategy - MEDLINE/PubMed. (DOCX 19 kb) [file 13643_2018_788_MOESM2_ESM.docx]

**Additional file 2: Example Search Strategy - MEDLINE/PubMed**

|  | **AND** |  | **AND** |  | **AND** |  | **NOT** |  | **NOT** |  |
| --- | --- | --- | --- | --- | --- | --- | --- | --- | --- | --- |
| Breast milk* [ALL] |  | Breast feeding [MH] |  | Macronutrient* [ALL] |  | English[lang] |  | Animals [MH] NOT Humans [MH] |  | HIV [MH] |
| Breastmilk* [ALL] |  | Breast milk express* [ALL] |  | Lactose [ALL] |  |  |  |  |  | pollutant [ALL] |
| Milk, Human [MH] |  | Breastmilk express* [ALL] |  | Proteins [MH] |  |  |  |  |  |  |
| Human milk* [ALL] |  |  |  | Protein [ALL] |  |  |  |  |  |  |
| Lactation” [ALL] |  |  |  | Proteins [ALL] |  |  |  |  |  |  |
|  |  |  |  | Lipids [MH] |  |  |  |  |  |  |
|  |  |  |  | Lipid [ALL] |  |  |  |  |  |  |
|  |  |  |  | Lipids [ALL] |  |  |  |  |  |  |

((((((Breast milk* [ALL] OR Breastmilk* [ALL] OR Milk, Human [MH] OR Human milk* [ALL] OR “Lactation” [ALL]) AND (Breast feeding [MH] OR Breast milk express* [ALL] OR Breastmilk express* [ALL]) AND (Macronutrient* [ALL] OR Lactose [ALL] OR Proteins [MH] OR Protein [ALL] OR Proteins [ALL] OR Lipids [MH] OR Lipid [ALL] OR Lipids [ALL]) AND English[lang] NOT (Animals [MH] NOT Humans [MH]) NOT (HIV [MH] OR pollutant [ALL]))))))

Filters: “Humans” (species); “English” (languages)
